# Investigating new opportunities to combat stigma in healthcare through a “universal” approach: findings from a longitudinal study of healthcare workers in Mombasa, Kenya

**DOI:** 10.3389/fpubh.2026.1910801

**Published:** 2026-09-24

**Authors:** Ahmed Adam, Patricia Owira, Nick Olsen, David Kirongo, Grace Mwendar, Makobu Kimani, Kelly Khabala, Hakima Masud, Celina Kithinji, Masha Pastrana, Adrian Guadarrama, Imani Blessings, Thomas Omache, Timothy Broady, Iza Ciglenecki, Denton Callander

**Affiliations:** 1 Mombasa County Department of Health, Mombasa, Kenya; 2 International Center for Reproductive Health Kenya, Mombasa, Kenya; 3 Stats Central, Mark Wainwright Analytical Centre, University of New South Wales, Sydney, NSW, Australia; 4 Médecins Sans Frontières Mombasa Project, Mombasa, Kenya; 5 Kenya Medical Research Institute, Kilifi, Kenya; 6 Médecins Sans Frontières Switzerland, Geneva, Switzerland; 7 Hope Trans Initiative, Mombasa, Kenya; 8 Youth Advisory Champions for Health, Mombasa, Kenya; 9 University of New South Wales, Sydney, NSW, Australia

**Keywords:** adolescents, key populations, Mombasa, stigma, structural equation modeling, universal precaution, young people

## Abstract

Stigma plays a significant role in health and healthcare. To date, anti-stigma efforts have primarily focused on specific individual domains (e.g., mental health, HIV), resulting in fragmented efforts to address many different “stigmas.” Theorists now advocate for shifting toward a universal framework to understand and respond to stigma. To empirically test the potential utility of stigma as a universal concept, we analyzed data from 453 healthcare workers participating in a prospective cohort in Mombasa, Kenya. Across four waves comprising 925 points of data (June 2023–December 2024), we found strong correlations between all stigma domains. For example, stigma toward abortion was positively correlated with stigma toward mental health and vice versa (*ρ* = 0.53, 95% CI: 0.47, 0.56). Using structural equation modeling, we tested two models for organizing stigma domains. In the best-fitted model, stigma domains were organized into two thematic categories: conditions & experiences (mental health, abortion, sexual violence, early pregnancy) and behaviors & identities (sex work, drug use, sexual and gender minorities). These two domains were then further organized into stigma as an overarching latent variable, with strong independent associations (*β* = 0.65, 95% CI: 0.57, 0.73 and *β* = 0.61, 95% CI: 0.54, 0.68, respectively). Further, stigma as a latent variable was independently associated with healthcare workers’ individual dispositions, including agreeableness (*B* = −0.19, 95% CI: −0.34, −0.05) and acceptance of others (*B* = −0.28, 95% CI: −0.39, −0.18). These results support stigma as a “universal” heuristic among healthcare workers. Anti-stigma interventions should reduce domain-specific activities toward a “universal precautions” approach targeting larger worldviews, which can improve scalability and resource-efficiency.

## 1 Introduction

Stigma enacted within healthcare is a pervasive and powerful force, which some have defined as “a fundamental cause of population health inequalities” (1). While there are many proposed definitions, most build upon Goffman’s seminal idea of stigma as an unfavorable judgment applied to a group of people based on stereotypes or characteristics that are not inherently immoral or negative (2, 3). Social oppression is, reflexively, both the result and source of this stigma, which equates to diminished political, legal, social, and other forms of power (4). Although stigma as a concept was first introduced in the 1960s, it has gained significant purchase within the field of public health primarily in the last two decades. The widespread problem of stigma in healthcare settings is understood to affect people’s ability to access care and their health outcomes (5).

In health systems, stigma can manifest internal, interpersonal, and structural levels (6, 7). Numerous studies from around the world find that stigma expressed on any of these levels is detrimental to individual and public health, including direct effects like impeding access, reducing retention, damaging trust, harming mental health, and diminishing treatment outcomes (e.g., 1, 5, 8–11). To date, most of the research on stigma has focused on specific individual domains. Among the most prominently studied forms of stigma relate to HIV (12, 13) and mental health (14, 15), but certainly there have been numerous others, including stigma toward obesity (16), sexual violence (17), abortion (18), diabetes (19), drug use (20), and many others. This list is by no means comprehensive; indeed, one study identified 93 different domains of stigma described in the extant health literature (21).

With growing recognition of stigma’s impact on health, so has there been considerable investment in anti-stigma intervention globally. As with studies articulating experiences and expressions of stigma, advocacy and other interventions have largely been siloed within specific domains of focus. For example, ending stigma toward people with mental health conditions was the primary focus of a recent Lancet Commission (22) and is a clearly stated goal of the World Health Organization (23) and the Kenyan Ministry of Health (24). To that end, there have been dozens of studies investigating interventions to reduce stigma in the domain of mental health (Gronholm et al., 2017; Thornicroft et al., 2016), (25). Similarly, ending stigma in relation to HIV is a cornerstone of policy in Kenya (26) and internationally (27), and the focus of dozens of intervention studies worldwide (e.g., 12, 13, 28, 29).

Our attention to mental health and HIV stigma is not to single out these areas especially, but instead to demonstrate how efforts to address stigma in public health have relied heavily on domains to establish boundaries of study, intervention, funding, and policy (30). In an article detailing what they call “the stigma complex”, Pescosolido and Martin contend that this approach has created “increasingly specific and multidimensional definitions” of stigma and associated intervention (30). Relatedly, in recent years there has been greater attention to intersectional stigma, which recognizes how multiple experiences of oppression ‘intersect’ to create mutually constituting and unique stigma positionalities (31–33). Thus, it has become common practice in stigma research and intervention to focus on narrowly defined and highly specific domains.

While there is certainly great value in understanding the intricacies of stigma across diverse domains, populations, and other contextual factors, working exclusively within a domain-specific framework may impede effective intervention in healthcare (34). As noted, one study found over 90 different stigmatized domains referenced in the public health literature (21); within the current domain-focused approach to anti-stigma intervention, it is difficult to see how targeting each of these and their many possible intersectional combinations could ever be meaningfully accomplished. Another limitation of a domain-specific approach is that some tend to be prioritized over others. For example, one study of low-and-middle-income countries found that 78% of all anti-stigma interventions were focused on HIV or mental health, with other domains receiving little or no attention (25). Further, the global healthcare workforce—especially in low-and-middle-income countries like Kenya—is overextended and facing increasing shortages of personnel (35). To imagine that time-poor healthcare workers can effectively engage with, retain, and act upon numerous domain-specific anti-stigma programs is simply not realistic.

One promising alternative to traditional domain-focused stigma scholarship and intervention comes from Treloar and colleagues who propose centering such work within what they call a “universal precautions framework” (34). The premise of this framework is that stigma is something “universal” that we all experience for some reason and at some point, which is supported by research findings that over 95% of people identify with one or more stigmatized experiences (21). Approaching stigma as a universal experience elevates our sight beyond its specific manifestations to gaze more broadly at its overarching qualities and implications, which may also foster more holistic, encompassing, and, hopefully, effective anti-stigma intervention (34). Overall, this idea re-establishes stigma as the overarching theoretical framework, which aside from being conceptually logical is also rather practical. As Treloar and colleagues contend: “Expecting the health system…to respond with equal and unique attention to each of our very important agendas is impractical and unrealistic” (34).

While a universal precautions anti-stigma framework may seem promising, empirical work is needed to test and validate its central assumptions. Specifically, we need to investigate if stigmas manifesting across diverse domains form cohesive relationships. Given the fragmentation of stigma work over the last 20 years, it is quite possible that what stigma means in one domain is very detached from what it has come to mean in others (30). If stigma can indeed be understood from a universal perspective, then we would expect that stigma in a specific domain relates to stigma in others, and that all domains can be aggregated up to a single overarching concept of “stigma”.

To undertake these investigations and test the empirical validity of a “universal” approach to stigma scholarship and intervention, we analyze data from a longitudinal study of healthcare workers in Mombasa, Kenya known as the Mombasa Healthcare Worker Survey.

## 2 Methods

### 2.1 Study design

A longitudinal cohort was established comprising repeated surveys of healthcare workers in Mombasa, Kenya. This analysis pertains to four waves of baseline data collected from June 2023 to December 2024. Wave 1 was conducted in June 2023, Wave 2 in November 2023, Wave 3 in September 2024, and Wave 4 in December 2024. The extended period between Wave 2 and Wave 3 was owing to a healthcare worker strike that took place across Kenya in mid-2024.

### 2.2 Study setting

The study took place at six health services located in Mombasa County, which is Kenya’s second most populous city. Three study sites were selected because they were participating in an intervention to improve healthcare for adolescents and young people, while the remaining three were selected in consultation with the local health department to serve as matched “control” sites (i.e., those not receiving the intervention). All sites were classified as “level 2” facilities within the Kenyan public health system, which refers to those that offer basic outpatient care (36). The timeframe analyzed here is the baseline before any anti-stigma programming was implemented at intervention sites.

### 2.3 Study participants

To be eligible, participants had to be 18 years or older, employed by a participating health service, and able to read English or Swahili. All clinic employees were eligible, including those who provided direct patient care (e.g., nurses, doctors, psychologists) and others (e.g., security guards, health record managers).

### 2.4 Data collection

Data was collected via de-identified repeat digital surveys administered once per study wave. The survey included items on participant sociodemographic characteristics, work experience, and stigmatizing attitudes toward seven domains. Given the larger intervention’s focus on adolescents and young people, the specific domains assessed were chosen in collaboration with communities of healthcare workers, public health officials, and young people themselves.

Existing stigma measures were used, with priority for those tailored for use in Kenya and/or the healthcare sector. Measured stigmas included toward people with mental health challenges (Modgill et al., 2014), young mothers (Hall et al., 2018), survivors of sexual violence (Thelan and Meadows, 2022), those who have had an abortion (Makenzius et al., 2019), sex workers, people who use drugs, and sexual and gender minorities (37). Some healthcare-specific items were also asked (e.g., “I would be willing to offer medical care to a sex worker”), with survey routing ensuring they were shown only to clinical participants; responses were scored on a five-point Likert scale from “strongly disagree” to “strongly agree”. These domains of stigma were selected for measurement because they were identified via collaboration with young people in Mombasa as particularly relevant to their experiences of health and healthcare.

Beyond direct measures of stigma, we also used established measures to assess various participant characteristics, which may have been related to reporting or expressions of stigma. Social desirability was assessed using a short form of the Marlowe-Crowne Social Desirability Scale (38). In Wave 4, the personality trait “agreeableness” was assessed using a short version of the Big Five Inventory (39), while the trait “acceptance” was assessed using the Acceptance of Others Scale (40). These items were added only in Wave 4 to assess new ideas introduced over time by the investigative team and our collaborators.

This study was conducted by institutions who could have been perceived as having authority over participating healthcare workers. Thus, the study design prioritized anonymity and did not uniquely identify participants across study waves. Participants were, however, asked if they had completed the survey prior, which was used to estimate the number of individuals in the sample.

### 2.5 Variables

Participant details were organized in terms of age, gender (men, women, non-binary), level of education, and length of time in current position. Participants were also organized in terms of their work type (clinical, non-clinical), study site, and wave of participation. Scores for all measures were calculated as per the established standards for each scale. All stigma scale scores were standardized as an average across items, creating a possible range of 1 (very low stigma) to 5 (very high stigma). The use of a standardized average enable comparisons between different stigma domains and participant groups.

### 2.6 Analyses

To assess internal reliability for this context and our sample, Cronbach’s *α* was calculated for each established measure. We also conducted bivariable and bidirectional correlation analyses to compare relationships between all measures of stigma. To assess potential clustering effects, we analyzed stigma results between study sites using bivariable linear regression. As noted, participant data were not linked between waves; to partially account for this issue, wave was fixed as an independent variable in all analyses and heteroskedasticity-robust standard errors were deployed. To account for the potential of socially desirable reporting to influence our primary outcomes, scores on the Marlow-Crowe Social Desirability Scale were included as covariates in all analyses.

To investigate the suitability of a “universal” conceptual framework for stigma and potential alternatives, two structural equation modeling (SEM) analyses were carried out. Model 1 included all measures of stigma within a single overarching latent variable of “stigma”. Model 2 investigated two potential latent variables (condition & experience, behavior & identity) and investigated their association with an overarching latent variable of “stigma” (Figure 1).

**Figure 1:**
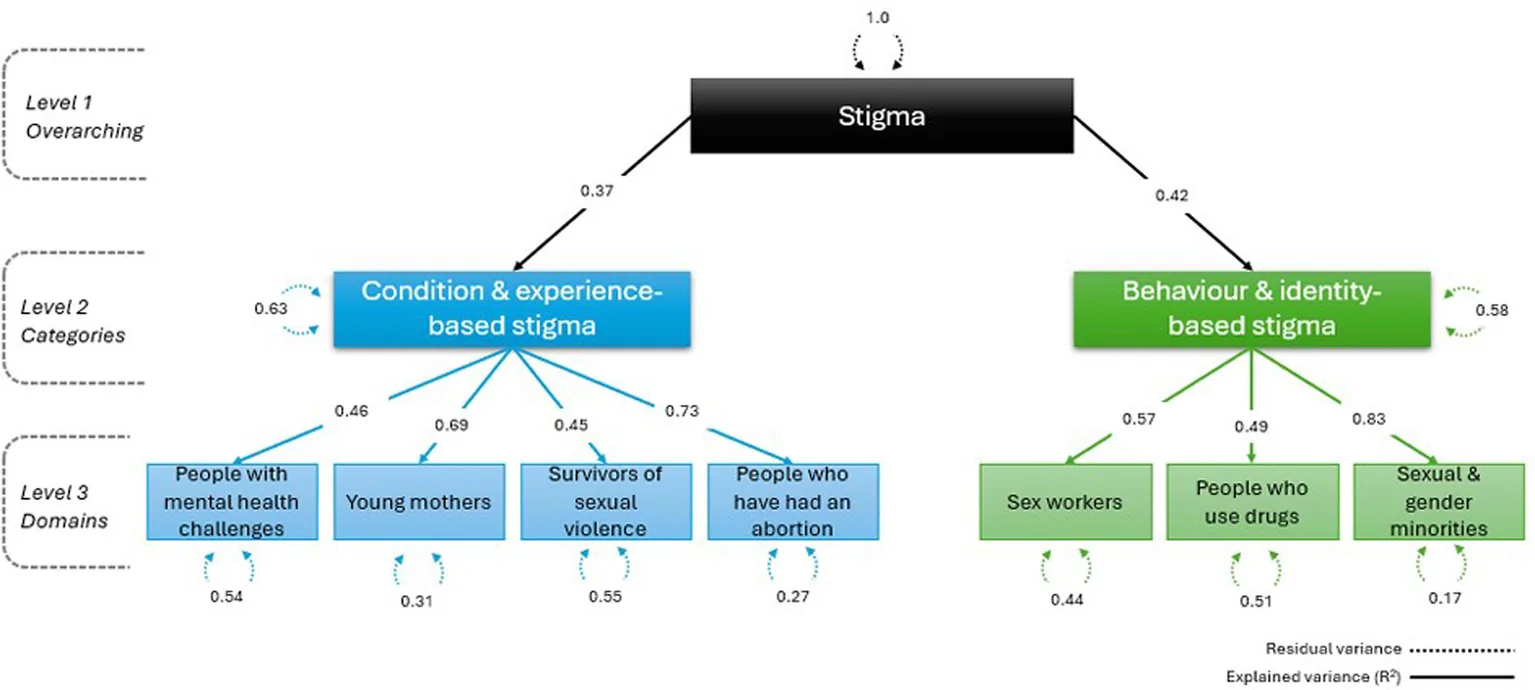
Conceptual framework and confirmatory factor analysis results (model 2), including explained and residual variance from higher-order latent variable structure.

Standard goodness of fit indicators was calculated for each model, including comparative fit index (CFI), Tucker-Lewis Index (TLI), root mean squared error of approximation (RMSEA), standardized root mean squared residual (SRMR), and coefficient of determination (CD). We also calculated the Akaike’s Information Criterion (AIC) and Bayesian Information Criterion (BIC) for each model. For ease of interpretation, results are presented as standardized *β* values with 95% confidence intervals (CI). These indicators were used to compare the models and make an overall assessment of their applicability to our conceptual framework. Given the use of repeated unlinked responses, we also conducted a sensitivity analysis constructing the same model, just among the sub-sample who reported no previous participation (i.e., first-time participants).

Finally, multivariable linear regression analyses were conducted to investigate demographic, professional, and personality factors associated with stigma as an overarching latent variable among the sample who participated in Wave 4, the final wave of the baseline period.

## 3 Results

### 3.1 Participant details

From June 2023 to December 2024, a total of 925 data points were collected representing 453 individual healthcare workers. There were 216 participants in Wave 1, 222 in Wave 2 (56.8% estimated repeat participation), 234 in Wave 3 (62.0% estimated repeat participation), and 253 in Wave 4 (73.1% estimated repeat participation). The number of individual participants per study site ranged from 49 to 110 with an average of 64.7 per site.

Individual participants ranged in age from 20 to 59 years (median = 35, interquartile range: 29–43.5) with a majority (71.1%) women. Most reported some form of education beyond high school (85.2%), and the majority were born in Kenya (98.7%). Four in five participants reported direct patient care (80.4%) while the remaining had non-clinical positions (19.6%). Regarding time in their position, 44.6% of participants had less than 2 years, 17.2% had 2–4 years, and 38.2% had four or more years. Full sample details are provided in Table 1.

**Table 1:** Sociodemographic characteristics of participants in the Mombasa healthcare worker survey (*n* = 453).

Characteristic	*n*	%
Age range (median; IQR)	20–59 (35; 29–43.5)	
Gender		
Women	314	69.32
Men	136	30.02
Non-binary	3	0.66
Highest level of education		
Primary or secondary	67	14.79
College/Trade school	226	49.89
University	160	35.32
Work type		
Clinical (direct patient care)	364	19.65
Non-clinical	89	80.35
Time in current position		
<2 years	202	44.59
2–4 years	78	17.22
≥4 years	173	38.19

### 3.2 Stigma domains

Figure 2 provides an overview of standardized scores on all stigma scales, including the mean scores. Cronbach’s *α* for each stigma measure ranged from 0.77–0.88 with a mean of 0.83 (SD: 0.03). On average, healthcare workers displayed the lowest levels of stigma toward young mothers (*M* = 1.95, 95% CI: 1.85, 2.06) and sex workers (*M* = 1.99, 95% CI: 1.90, 2.08) and highest levels of stigma toward women who had an abortion (*M* = 2.31, 95% CI: 2.21, 2.41) and people who use drugs (*M* = 2.48, 95% CI: 2.38, 2.59).

**Figure 2:**
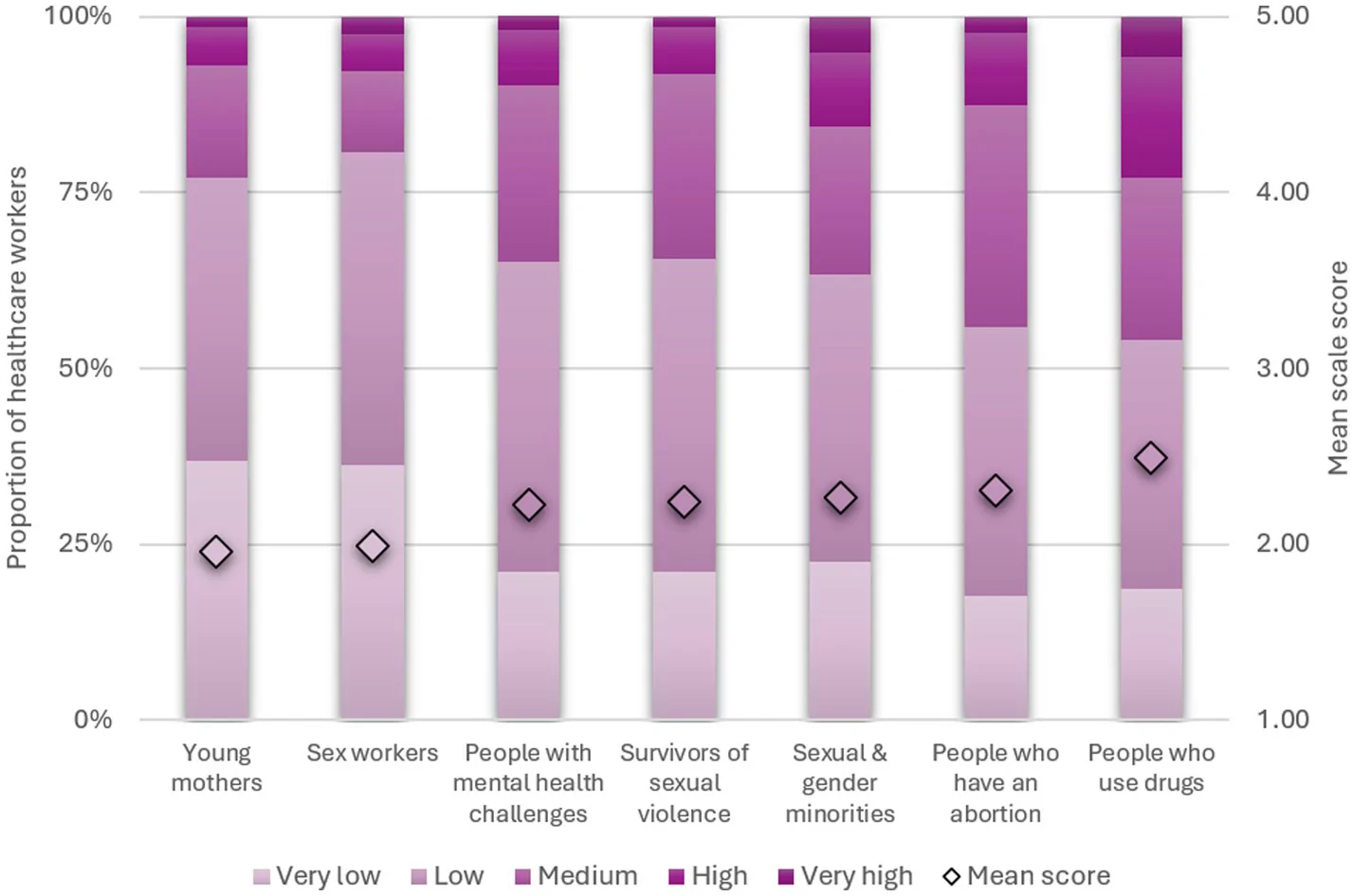
Levels of stigma among healthcare workers in Mombasa, by domain.

Within each domain, the proportion of healthcare workers reporting high stigma was generally low. The highest proportion overall was toward people who use drugs, with 22.9% of healthcare workers indicating “high” or “very high” stigma. However, two thirds of healthcare workers (69.7%) had high stigmas in at least one area. Indeed, 18.2% had high stigma in one domain, 17.6% in two domains, and 33.9% in three or more domains. A small minority (1.2%) had high stigma in every measured domain.

Stigma scores varied between study sites. For example, mean scores of mental health stigma ranged from 1.72–2.40 between sites, with an observable difference (*p* < 0.001) similar across domains. We observed no changes to any domain of stigma between baseline study waves. For example, the mean score of mental health stigma was 2.20 (Wave 1), 2.19 (Wave 2), 2.26 (Wave 3), and 2.25 (Wave 4). A test of trend found no observable change over time (*p* = 0.38), which was the same for other domains. All analyses, however, accounted for potential clustering by study site and wave by calculating the more cluster-robust (Huber-White) standard errors to allow for correlation of observations between sites and over time while maintaining independence between observations.

### 3.3 Stigma relationships

Figure 3 details the bivariable and bidirectional relationships between each measure of stigma. We observed strong and positive two-way Spearman’s correlations for all combinations of stigma, meaning that increasing stigma in one domain was associated with increasing stigma in all others. For example, stigma toward early pregnancy was strongly and positively correlated with stigma toward abortion, and vice versa (*ρ* = 0.70, 95% CI: 0.66, 0.74).

**Figure 3:**
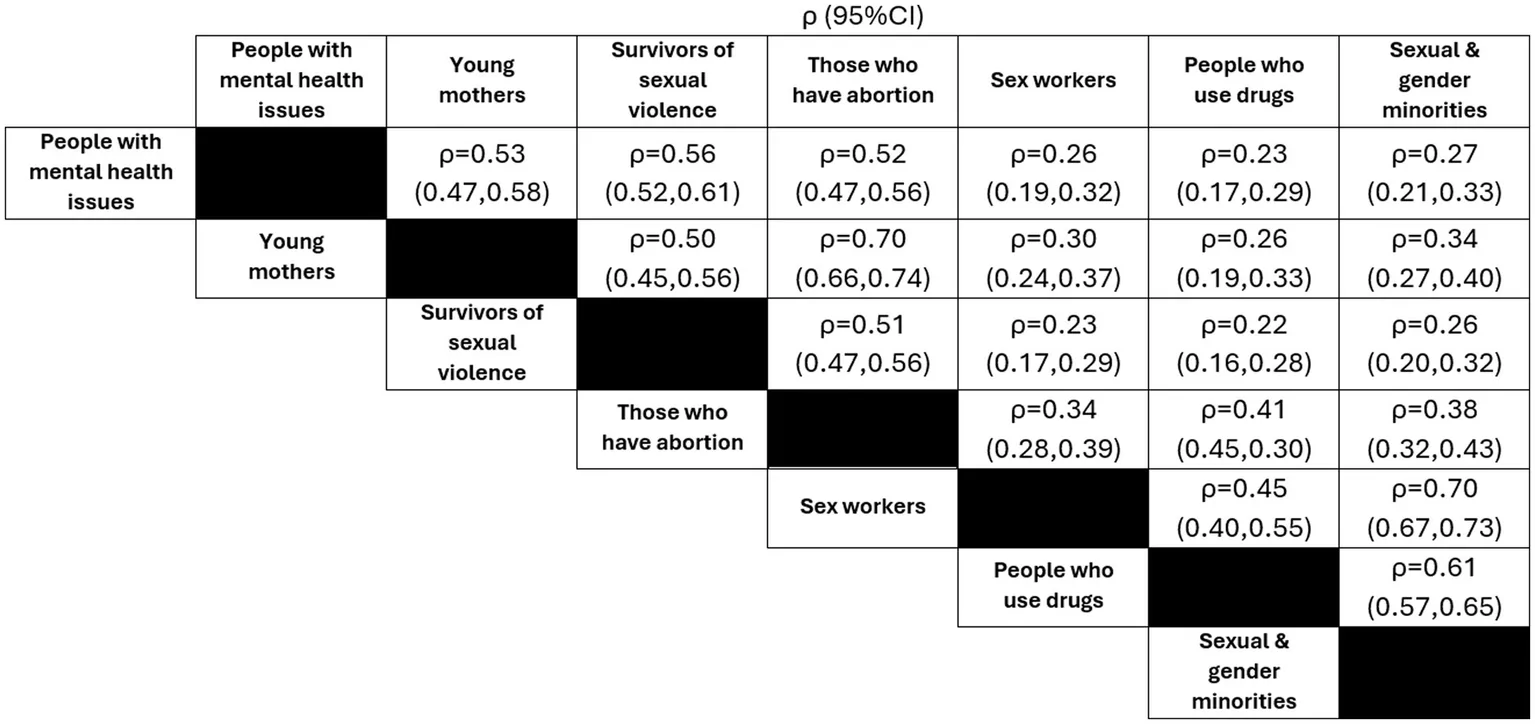
Spearman correlation analyses of bivariable associations between various stigma domains among healthcare workers in Mombasa. CI, confidence interval.

Table 2 details the results of our two SEM models. In the first SEM model, all domain-specific expressions of stigma were positively associated with “stigma” as an overarching latent variable. While the model explained a large amount of the observed variance (CD = 0.87), other indicators suggested poor fit. Notably, the RMSEA for this model was high (0.28, 95% CI: 0.26, 0.29) while the CFI and TLI were low (0.65 and 0.48, respectively). There was no difference to the model outcomes or fit when the model was clustered by study site.

**Table 2:** Comparing SEM outputs of stigma latent variables, including goodness of fit measures.

Stigma Domain	*β* (95% CI)	
	Model 1	Model 2

People with mental health challenges	0.67 (0.62, 0.79)	0.68 (0.63, 0.73)^a^
Young mothers	0.82 (0.79, 0.85)	0.83 (0.80, 0.86)^a^
Survivors of sexual violence	0.66 (0.61, 0.71)	0.67 (0.63, 0.72)^a^
People who have had an abortion	0.85 (0.82, 0.88)	0.85 (0.82, 0.88)^a^
Sex workers	0.41 (0.35, 0.48)	0.75 (0.71, 0.79)^b^
People who use drugs	0.36 (0.29, 0.43)	0.70 (0.65, 0.74)^b^
Sexual and gender minorities	0.41 (0.35, 0.48)	0.91 (0.88, 0.95)^b^
Categories (latent variables)		
Condition and experience	—	0.65 (0.57, 0.73)^c^
Behavior and identity	—	0.61 (0.54, 0.68) ^c^
Goodness of fit		
AIC	11612.80	10945.41
BIC	11708.61	11045.79
RMSEA (95% CI)	0.28 (0.26, 0.29)	0.10 (0.08, 0.12)
SRMR	0.16	0.04
CD	0.87	0.56
CFI	0.65	0.96
TLI	0.48	0.93

AIC, Akaike’s Information Criterion; BIC, Bayesian Information Criterion; RMSEA, root mean squared error of approximation; SRMR, standardized root mean squared residual; CD, coefficient of determination; CFI, comparative fit index, TLI, Tucker-Lewis Index.

a In this model, organized into “condition and experience” latent variable.

b In this model, organized into “identity and behavior” latent variable.

c As related to the higher-order latent variable “stigma” (only included in Model 2).

Results of the second SEM model are also shown in Table 2. Drawing upon previous conceptual work (41), the first factor (“condition & experience”) comprised stigma toward people with mental health challenges, young mothers, those who have had an abortion, and survivors of sexual violence. The second factor (“behavior and identity”) comprised stigma toward sex workers, people who use drugs, and sexual and gender minorities. Each domain of stigma (Level 3) was positively associated with a latent category of stigma (Level 2), and each category was positively associated with stigma as an overarching concept (Level 1). Specifically, stigma based on condition & experience was positively associated with stigma overall (*β* = 0.65, 95% CI: 0.57, 0.73) as was stigma toward behavior & identity (*β* = 0.61, 95% CI: 0.54, 0.68). Figure 1 provides full details of the explained and unexplained variance in this model. Comparatively, the second model indicated better fit than the first, suggesting stigma can be understood as an overarching experience but with some internal distinctions. Further, results from the sensitivity analysis found the SEM models were essentially the same overall and compared just to each healthcare worker’s first time participating in the study; full details are provided in the Supplementary Table S1.

### 3.4 Overarching stigma and associated factors

Using the SEM results, a latent factor score (“overarching stigma”) was created for each participant, which was then used in two subsequent regression analyses to investigate associated factors. The first regression analysis explained 23.2% of the observed variance in overall stigma. Age was negatively associated with stigma (*B* = −0.003, 95% CI: −0.01, −0.001), while men had higher stigma compared to women (*B* = 0.06, 95% CI: 0.01, 0.10). Non-binary participants had lower levels of stigma than women (*B* = −0.21, 95% CI: −0.28, −0.05), noting the small number included in our sample (*n* = 6). Compared to healthcare workers with primary or secondary education, stigma was lower among those with college or vocational training (*B* = −0.13, 95% CI: −0.19, −0.07) and even lower among those with a university background (*B* = −0.19, 95% CI: −0.25, −0.13). Table 3 provides all results of these analyses.

**Table 3:** Factors associated (multivariable linear regression) with stigma as an overarching variable among healthcare workers in Mombasa.

Factor	*Β* (95% CI)	
	Analysis 1 (*n* = 673)^a^	Analysis 2 (*n* = 251)^a^
Age (in years)	−0.003 (−0.01, −0.001)	0.001 (−0.004, 0.004)
Gender		
Women	[Ref]	[Ref]
Men	0.06 (0.01, 0.10)	0.04 (−0.03, 0.12)
Non-binary	−0.21 (−0.28, −0.05)	0.10 (−0.02, 0.22)
Highest level of education		
Primary or secondary	[Ref]	[Ref]
College/trade school	−0.13 (−0.19, −0.07)	−0.10 (−0.18, −0.02)
University	−0.19 (−0.25, −0.13)	−0.13 (−0.22, −0.03)
Work type		
Non-clinical	[Ref]	[Ref]
Clinical (patient care)	−0.04 (−0.09, 0.01)	−0.01 (−0.12, 0.11)
Time in current position		
<2 years	[Ref]	[Ref]
2–4 years	−0.01 (−0.04, 0.06)	−0.013 (−0.11, 0.09)
≥4 years	0.06 (0.01, 0.11)	0.01 (−0.08, 0.09)
Personality characteristics		
Social desirability	−0.03 (−0.05, −0.01)	^c^
Agreeableness	^b^	−0.09 (−0.15, −0.03)
Acceptance of others	^b^	−0.14 (−0.20, −0.09)

a Sample size refers to the number of data points, not individual participants; study wave accounted for the analysis alongside robust standard errors used.

b These scales were only introduced in Wave 4 and, therefore, not included in Analysis 1.

c This scale was removed in Wave 4 and, therefore, not included in Analysis 2.

The second analysis incorporated measures of personality introduced in Wave 4. This regression captured 32.8% of the observed variance in stigma. Agreeableness was negatively associated with stigma (*B* = −0.09, 95% CI: −0.15, −0.03) as was acceptance of others (*B* = −0.28, 95% CI: −0.39, −0.18; *β* = −0.33). In this second analysis, when controlling agreeableness and acceptance, only level of education remained associated with levels of stigma measured among healthcare workers.

## 4 Discussion

The results of this study show that stigma is common among healthcare workers in Mombasa, with two thirds indicating high stigma toward at least one topic domain. Although stigma was expressed within specific domains (e.g., mental health), such domains were strongly correlated and could be organized into larger categories defined by condition, experience, behavior, and identity. Importantly, we observed that domains of stigma could be further organized into a single overarching concept of stigma writ large. We also found preliminary evidence that overarching stigma was associated with personality characteristics like agreeableness and acceptance of others.

Our results offer empirical support for the conceptualization of stigma as a universal yet multidimensional construct within healthcare settings. The observed differences between various similar but distinct conceptual organizations are instructive. Notably, while our first model posited a single latent factor of stigma and did find strong associations between individual domains and an overarching construct, ultimately this unidimensional model had poor fit to the data. This finding suggests that while healthcare workers’ stigmatizing beliefs are strongly related, they are not easily reducible to a single conceptual dimension.

By comparison, introducing second-order categories of stigma fit much more closely to the data. Specifically, stigmas toward people with mental health challenges, young mothers, survivors of sexual violence, and those who have had an abortion could be grouped under a category of “condition and experience”, while stigma toward sex workers, people who use drugs, and sexual and gender minorities could be grouped as “behavior and identity”. Such clusters suggest a difference in how healthcare workers assign judgment to various forms of so-called social deviance, although our data did not allow investigation of why these categories form. These results align with previous conceptual efforts to organize stigma (41), and they signal some utility in grouping individual stigmas into broader categories. An expanded list of stigma domains and other measurements may yield other groupings.

Importantly, we observed that groupings of stigma domains were ultimately associated with an overarching heuristic stigma. This result supports the idea of stigma as a concept that binds together diverse expressions of prejudice, which aligns with a “universal” theory of stigma in healthcare as posited by Treloar and colleagues (34). As noted, however, simply grouping all stigma domains into a single overarching concept was not well fitted to our data, suggesting such a formulation may obscure potential mechanisms of prejudice. Taken together, our results suggest the most robust approach is one incorporating some distinctions between various stigmas situated within a universal framework.

We found education was strongly and inversely associated with stigma among healthcare workers, including when accounting for other individual differences. This result is consistent with previous studies, which found higher levels of formal education were associated with lower levels of various stigmas (42–44). This association may reflect the role of education in fostering critical thinking, empathy, and cognitive flexibility, all of which are foundational for reducing reliance on moral heuristics or social stereotypes. It also signals the significant potential value of education toward anti-stigma intervention.

Additionally, our findings shed light on potential relationships between personality dispositions and stigmatizing beliefs. Specifically, healthcare workers with higher scores in agreeableness and acceptance of others had lower stigmas across domains. Importantly, when agreeableness and acceptance were included in our multivariable models, the previously observed associations with demographic variables (notably gender and age) were attenuated. Such findings align with the limited extant literature on disposition and stigma (45, 46), and in many ways they are logically apparent. They also lend further support to the idea of “universal precautions” to combat stigma; it may be that promoting generalist ideals like acceptance and agreeableness is more effective than engaging with domain-specific concerns.

Our findings offer implications for future intervention. First, they suggest possibilities of reorienting anti-stigma healthcare interventions away from a domain-specific focus to higher-order and universal approaches. As noted, others have advocated for this need and our study lends empirical support for such change (21, 30, 34). Moving toward universal thinking in anti-stigma work also recognizes the practicalities of healthcare settings, notably resources and their restraints, which is an issue in Kenya and beyond. Thus, priority should be given to developing and evaluating stigma-reduction curricula that are not tethered to a particular domain but instead target the underlying mechanisms of stigma as a generalized phenomenon. Research to investigate the effectiveness of a ‘universal’ approach to anti-stigma intervention is warranted, including through comparison to more traditional vertical approaches.

This study is strengthened by its large and diverse sample of healthcare workers, high retention across four study waves, and rigorous statistical modeling. The use of SEM allowed robust testing of conceptual and theoretical models of stigma, while the inclusion of demographic and psychological predictors deepened our understanding of stigma’s social and cognitive foundations. It is also one of the few studies to examine many kinds of stigma at once, allowing a more holistic understanding of stigmas as interrelated rather than isolated. This integrative approach moves the field away from fragmented, condition-specific models and toward a system-thinking lens.

These results must be understood in the context of the study’s limitations. First, while the sample of healthcare workers in Mombasa is unique and relevant, it may not be representative of healthcare workers in other regions or countries. Future research should seek to replicate our findings in other contexts, including beyond health settings. Second, reliance on self-report measures may introduce social desirability bias particularly given the sensitive nature of these topics. As described, we attempted to control this in the analysis, but social desirability remains an unresolved limitation of this kind of research. Third, there is no perfect way to measure stigma. Although care was taken to identify and refine measures most relevant to the Kenya context, other measurements may yield different results. Fourth, although our model showed strong fit indices, there remained unexplained variance. Certainly, no model is ever exhaustive so future research should seek to identify different relationships including across other stigma domains not assessed here.

Collectively, these findings support a model of stigma that is universal in its frame while accommodating potential distinctions between the multitude of conditions, experiences, behaviors, and identities targeted by social oppression. Rather than viewing universality and specificity as competing logics, our results suggest they are complementary: stigma in healthcare is both widespread in its presence and structured in its expression. From this perspective, our results support a more integrated, consolidated, system-wide, and ‘universal’ approach to anti-stigma intervention in health systems.

## 5 Conclusion

This study demonstrates that stigma among healthcare workers extends beyond individual conditions, experiences, behaviors, or identities and instead reflects a broader, interconnected phenomenon. Although healthcare workers expressed varying levels of stigma across different domains, strong correlations between all forms of stigma suggest that prejudicial attitudes are often shared across multiple populations and experiences. Structural equation modeling further revealed that while stigma can be organized into meaningful categories namely conditions and experiences, and behaviors and identities these categories are ultimately linked through a higher-order stigma construct. These findings provide empirical support for conceptualizing stigma as a universal yet multidimensional phenomenon within healthcare settings.

The study also highlights the importance of individual dispositions in shaping stigmatizing attitudes. Higher levels of education, agreeableness, and acceptance of others were associated with lower overall stigma, suggesting that stigma is influenced not only by knowledge of specific populations but also by broader values and worldviews. Together, these findings support a shift away from fragmented, domain-specific anti-stigma interventions toward more integrated approaches that address the underlying drivers of prejudice and discrimination. Such an approach may be particularly valuable in resource-constrained health systems where healthcare workers face competing demands and limited opportunities for repeated, condition-specific training.

## 6 Recommendations

The findings support the adoption of a universal stigma reduction framework within healthcare systems. Rather than implementing separate anti-stigma interventions for HIV, mental health, sexual and reproductive health, substance use, and other domains, policymakers and health program managers should develop integrated training and service delivery approaches that address the common underlying mechanisms of stigma. Such approaches should emphasize empathy, respect, acceptance of diversity, and person-centered care while recognizing the unique manifestations of stigma across different populations. Integrating stigma reduction into existing healthcare worker training, quality improvement initiatives, and routine supervision structures may improve efficiency, sustainability, and scalability.

Future efforts should also focus on strengthening educational and organizational environments that foster inclusion and acceptance. Healthcare worker curricula, continuing professional development programs, and workplace interventions should incorporate values clarification, reflective practice, and interpersonal skills development to address broader attitudes that contribute to stigma. Further research is needed to validate the universal stigma framework in other settings, explore additional stigma domains, and evaluate whether universal anti-stigma interventions are more effective and cost-efficient than traditional domain-specific approaches. By addressing stigma as a cross-cutting health system issue, healthcare programs can contribute to more equitable, accessible, and person-centered care for all populations.

## Data Availability

The de-identified data used to generate this analysis will be made available by the authors, without undue reservation upon request.
