# Correction: Investigating new opportunities to combat stigma in healthcare through a “universal” approach: findings from a longitudinal study of healthcare workers in Mombasa, Kenya

**DOI:** 10.3389/fpubh.2026.2019690

**Published:** 2026-10-15

**Authors:** Frontiers Production Office

**Affiliations:** Frontiers Media SA, Lausanne, Switzerland

**Keywords:** adolescents, key populations, Mombasa, stigma, structural equation modeling, universal precaution, young people

Affiliation University of New South Wales, Sydney, NSW, Australia was omitted from the paper. This affiliation has now been added for author(s) Timothy Broady and Denton Callander.

Affiliation Stats Central, Mark Wainwright Analytical Centre, University of New South Wales, Sydney, NSW, Australia was erroneously given as Stats Central, Mark Wainwright Analytical Centre, Sydney, NSW, Australia.

The correct affiliation list appears below:

Ahmed Adam^1*^, Patricia Owira^2^, Nick Olsen^3^, David Kirongo^4^, Grace Mwendar^4^, Makobu Kimani^5^, Kelly Khabala^4^, Hakima Masud^4^, Celina Kithinji^1^, Masha Pastrana^6^, Adrian Guadarrama^6^, Imani Blessings^7^, Thomas Omache^8^, Timothy Broady^9^, Iza Ciglenecki^6^ and Denton Callander^4, 9^

^1^Mombasa County Department of Health, Mombasa, Kenya

^2^International Center for Reproductive Health Kenya, Mombasa, Kenya

^3^Stats Central, Mark Wainwright Analytical Centre, University of New South Wales, Sydney, NSW, Australia

^4^Médecins Sans Frontières Mombasa Project, Mombasa, Kenya

^5^Kenya Medical Research Institute, Kilifi, Kenya

^6^Médecins Sans Frontières Switzerland, Geneva, Switzerland

^7^Hope Trans Initiative, Mombasa, Kenya

^8^Youth Advisory Champions for Health, Mombasa, Kenya

^9^University of New South Wales, Sydney, NSW, Australia

The original version of this article has been updated.

