# Supplementary material for: Investigating new opportunities to combat stigma in healthcare through a “universal” approach: findings from a longitudinal study of healthcare workers in Mombasa, Kenya
Source: Front Public Health. 2026 Sep 24;14:1910801. doi: 10.3389/fpubh.2026.1910801 (PMC13645603; doi:10.3389/fpubh.2026.1910801)
Supplement: Supplementary file 1 [file Table_1.docx]

**SUPPLEMENTARY APPENDIX**

**Table S1.** Sensitivity analysis comparing SEM results across all data points and just at each participant’s first time completing the survey

| **Stigma Domain** | **β (95%CI)** | |
| --- | --- | --- |
|  | **All data points**  (n=925) | **First data point**  (n=453) |
|  |  |  |
| People with mental health challenges | 0.68 (0.63, 0.73) ^a^ | 0.66 (0.56-0.76) ^a^ |
| Young mothers | 0.83 (0.80, 0.86) ^a^ | 0.79 (0.71-0.86) ^a^ |
| Survivors of sexual violence | 0.67 (0.63, 0.72) ^a^ | 0.67 (0.57-0.76) ^a^ |
| People who have had an abortion | 0.85 (0.82, 0.88) ^a^ | 0.76 (0.68-0.83) ^a^ |
| Sex workers | 0.75 (0.71, 0.79) ^b^ | 0.77 (0.70-0.84) ^b^ |
| People who use drugs | 0.70 (0.65, 0.74) ^b^ | 0.66 (0.57-0.74) ^b^ |
| Sexual & gender minorities | 0.91 (0.88, 0.95) ^b^ | 0.92 (0.86-0.98) ^b^ |
| **Categories (latent variables)** |  |  |
| Condition & experience | 0.65 (0.57, 0.73) | 0.67 (0.83-0.82) |
| Identity & behaviour | 0.61 (0.54, 0.68) | 0.61 (0.48-0.73) |
| **Goodness of fit** |  |  |
| AIC | 10945.41 | 3686.39 |
| BIC | 11045.79 | 3762.59 |
| RMSEA (95%CI) | 0.10 (0.08, 0.12) | 0.10 (0.07-0.14) |
| SRMR | 0.04 | 0.05 |
| CD | 0.56 | 0.58 |
| CFI | 0.96 | 0.95 |
| TLI | 0.93 | 0.92 |

*AIC=Akaike’s Information Criterion, BIC=Bayesian Information Criterion, RMSEA=Root Mean Squared Error of Approximation, SRMR=Standardized Root Mean Squared Residual, CD=Coefficient of Determination, CFI=Comparative Fit Index, TLI=Tucker-Lewis Index*

a. *In this model, organized into ‘condition & experience’ latent variable*
b. *In this model, organized into ‘identity & behaviour’ latent variable*
